# Antenatal corticosteroid administration in women undergoing tocolytic treatment who delivered before 34 weeks of gestation: a retrospective cohort study using a national inpatient database

**DOI:** 10.1186/s12884-019-2174-1

**Published:** 2019-01-09

**Authors:** Daisuke Shigemi, Hideo Yasunaga

**Affiliations:** Department of Clinical Epidemiology and Health Economics, School of Public Health, The University of Tokyo, 7-3-1, Hongo, Bunkyo-ku, Tokyo, 1130033 Japan

**Keywords:** Betamethasone, Dexamethasone, Preterm birth, Ritodrine, Tocolysis

## Abstract

**Background:**

Antenatal corticosteroid treatment is globally recommended for women at risk of giving birth before 34 weeks of gestation. In Japan, data on the rate of completing recommended antenatal corticosteroid treatment are lacking. This study aimed to: (i) determine the proportion of patients treated for threatened preterm birth with tocolysis who received antenatal glucocorticoids; and (ii) analyze the association between long-term tocolysis and antenatal glucocorticoids treatment as recommended.

**Methods:**

This was a retrospective cohort study using a national inpatient database in Japan. We selected pregnant women who had undergone treatment in hospitals due to threatened preterm birth and received the tocolytic ritodrine hydrochloride by infusion from July 2010 to March 2016, and delivered at < 34 weeks of gestation. The primary outcome was receiving of antenatal glucocorticoid treatment as recommended. Multivariable logistic regression was performed to evaluate factors associated with receiving antenatal glucocorticoid treatment.

**Results:**

Only 23% of 4048 eligible patients received glucocorticoid treatment as recommended. Those with longer durations of ritodrine hydrochloride infusion were significantly less likely to receive glucocorticoid treatment as recommended.

**Conclusions:**

In Japan, many patients who receive tocolytic treatment for threatened preterm birth do not receive antenatal glucocorticoid treatment as recommended. Recommended treatment based on apparent evidences should be performed for the patients with threatened preterm birth.

## Background

Preterm babies have an increased risk of serious illness or death during the neonatal period. Without appropriate treatment, babies born preterm are at increased risk of lifelong disability and poor quality of life [1, 2]. Respiratory morbidity including respiratory distress syndrome is a serious complication of preterm birth and one of the most important cause of early neonatal mortality and disability. Intraventricular hemorrhage and necrotising enterocolitis are also severe complications of preterm birth.

Administration of antenatal corticosteroids is the most beneficial intervention for improving neonatal outcomes among patients who give birth preterm [3]. Extended tocolytic treatment for preterm labour is reportedly ineffective, whereas delay of delivery for 48 h in women with threatened labour before 34 weeks allows recommended antenatal corticosteroid treatment, which improves fetal maturity and enables transfer of the pregnant woman to a centre with a neonatal intensive care unit [4–7]. The prophylactic corticosteroids reduce the incidence of respiratory distress syndrome and, ideally, the glucocorticoid treatment in the recommended dosage should be completed within the 7 days before preterm birth [8]. A regimen of two doses of 12 mg of betamethasone given intramuscularly 24 h apart or four doses of 6 mg of dexamethasone given intramuscularly 12 h apart is recommended [9, 10]. In 2016, the American College of Obstetricians and Gynecologists’ Committee on Practice Bulletins strongly recommended a single course of corticosteroids for pregnant women between 24 weeks and 34 weeks of gestation who are at risk of delivery within 7 days [11]. This guideline also stated that a single repeat course of antenatal corticosteroids should be considered in women who are at less than 34 weeks of gestation, at risk of preterm delivery within the next 7 days, and whose prior course of antenatal corticosteroids was administered more than 14 days previously [11]. In addition, World Health Organization guidelines recommend administration of magnesium sulfate to women at risk of imminent birth before 32 weeks of gestation to prevent cerebral palsy [8].

There may be a gap between the available evidence and actual clinical practice for preterm labour in Japan. First, because the globally commonly administered medications for threatened preterm birth include nifedipine, atosiban, and vaginal progesterone and these agents are not covered by the universal healthcare coverage in Japan, they cannot be used for standard therapy in Japan. Second, some Japanese obstetricians are likely to select long-term (≥ 48 h) tocolysis with intravenous low-dose (50–200 μg/min) infusion of ritodrine hydrochloride, a betamimetic. In particular, long-term tocolysis is more likely to be used in hospitals without neonatal intensive care units. In addition, Japanese clinical guidelines do not recommend repeated courses of antenatal corticosteroids for women at risk of preterm birth [12, 13].

A major concern around this unscientific practice is the possible failure to administer maternal glucocorticoids as recommended before preterm birth. We hypothesized that physicians may not administer glucocorticoids in a timely manner because long-term tocolysis can mask deterioration in threatened preterm birth status. Additionally, there is no scientific data on the success rate of administration of appropriately timed glucocorticoids in Japan. Not only the success rate of administration of antenatal glucocorticoids, but the association between long-term tocolysis and rate of antenatal glucocorticoid administration could be important in improving fetal outcomes among patients who deliver before 34 weeks of gestation.

Using a national inpatient database in Japan, the present study aimed to determine the proportion of patients with threatened preterm birth who had received antenatal glucocorticoid treatment, including appropriately timed antenatal glucocorticoid treatment, during hospitalization. The association between long-term tocolysis and proportion of patients receiving glucocorticoids as recommended was also analysed.

## Methods

The Diagnosis Procedure Combination (DPC) database, a national inpatient database for acute-care inpatients in Japan, the details of which have been described elsewhere [14], was used in this retrospective cohort study. Briefly, about 1000 hospitals participate in the database and provide data for approximately eight million inpatient admissions annually, representing about 50% of all acute-care inpatients in Japan. The DPC database contains discharge abstracts and administrative reimbursement data for inpatient episodes supplied by the participating hospitals. The following data are included: dates of admission and discharge; patient age and sex; body weight and height; primary and secondary diagnoses; pre-existing comorbidities on admission and complications after admission; procedures performed; medications and devices used; in-hospital mortality; pregnancy status (pregnant or not); gestational age on admission, and delivery during hospitalization. Diagnoses, comorbidities, and complications are recorded using International Classification of Diseases Tenth Revision (ICD-10) codes and text data in Japanese. The database does not contain any laboratory data or findings on obstetric examination (including Bishop score, uterine cervical length, and vaginal bacteriological culture). The attending physicians are required to record the diagnoses accurately because the diagnostic records are linked to a payment system that is based on medical insurance. A previous study has shown that the validity of diagnostic records in the DPC database is generally high and that the sensitivity and specificity of the primary diagnoses are 78.9 and 93.2%, respectively [15].

Pregnant women aged 13–50 years who had been diagnosed with threatened preterm birth between 22 and 36 weeks of gestation (ICD-10 code, O470) and had undergone treatment in hospitals from July 2010 to March 2016 and for whom there was complete data were identified. From this pool, those who had deliveries < 34 weeks of gestation were finally selected as study patients.

Age was categorized into ≤19, 20–24, 25–29, 30–34, 35–39, and ≥ 40 years. The duration of ritodrine hydrochloride intravenous administration as tocolytic treatment was categorized into ≤48 h, 3–6 days, 7–13 days, 14–27 days, and ≥ 28 days. Patients who had received intravenous magnesium sulfate prior to administration of intravenous ritodrine hydrochloride were excluded because whether the magnesium sulfate had been administered for the purpose of tocolysis or to prevent cerebral palsy could not be determined for individual patients in the database.

We investigated the proportion of patients who received antenatal glucocorticoid as recommended, which was defined as administration of the recommended dosage of maternal glucocorticoids within 7 days before preterm birth (< 34 weeks of gestation) during hospitalization. Intramuscular injection of betamethasone 24 mg or dexamethasone 24 mg qualified patients for inclusion [9, 10]. We also investigated (i) the proportion of women who received the recommended dosage stratified by gestational age at delivery and tocolytic duration, (ii) administration of maternal glucocorticoids within 14 days before preterm birth (< 34 weeks of gestation), (iii) any administration of maternal glucocorticoids (both partial and complete) during hospitalization, (iv) timing of administration of maternal glucocorticoids after admission, (v) frequency of repeated courses 14 days or later from the first course of glucocorticoids, and (vi) intervals between the last glucocorticoid dose and preterm birth. Both timing of maternal glucocorticoids administration after admission and periods from the last glucocorticoids administration to preterm birth were presented using median and interquartile range.

Categorical variables (smoking, multiple births, premature rupture of membranes, placenta previa, hypertensive disorders of pregnancy, and gestational diabetes mellitus) were compared by the χ^2^ test or Fisher’s exact test. Continuous variables (age, body mass index, and gestational age at admission) were compared by a *t*-test or the Mann–Whitney U-test, as appropriate. Multivariable logistic regression analysis was performed to evaluate factors associated with administration of glucocorticoids as recommended. All statistical analyses were performed using IBM SPSS software version 23 (IBM, Armonk, NY, USA). All tests were two-tailed, and the threshold for significance was *P* < 0.05.

Written informed consent was not required because of the anonymous nature of the data. The need for written consent was formally waived by the Ethics Committee and the Institutional Review Board of The University of Tokyo approved the study.

## Results

During the study period, we identified 7298 hospitalized patients with a diagnosis of threatened preterm birth who had given birth before 34 weeks of gestation during hospitalization. Of these, 5197 patients had received intravenous ritodrine hydrochloride as tocolysis. We excluded 1149 of the 5197 patients because they had received intravenous magnesium sulfate prior to administration of intravenous ritodrine hydrochloride. The mean (standard deviation [SD]) age of the 4048 eligible patients was 31.5 (5.4) years, and the mean (SD) gestational age was 28.5 (3.4) weeks.

The 4048 patients’ characteristics according to duration of ritodrine hydrochloride infusion are shown in Table 1. The proportion of patients who had received tocolytic treatment for ≤48 h was only 36.4%, whereas 20.5 and 43.1% had received tocolytic treatment for 2–6 days and ≥ 7 days, respectively.

**Table 1:** Patient characteristics according to duration of ritodrine hydrochloride infusion

		Total	Duration of ritodrine hydrochloride infusion									
			≤48 h		3–6 days		7–13 days		14–27 days		≥28 days	
Number of patients		4048	1472	(36.4)	831	(20.5)	488	(12.1)	570	(14.1)	687	(17.0)
Age (years), mean (SD)	< 20	80	33	(41.3)	14	(17.5)	3	(3.8)	15	(18.8)	15	(18.8)
	20–24	358	137	(38.3)	77	(21.5)	40	(11.2)	53	(14.8)	51	(14.2)
	25–29	962	358	(37.2)	214	(22.2)	106	(11.0)	132	(13.7)	152	(15.8)
	30–34	1388	502	(36.2)	269	(19.4)	180	(13.0)	191	(13.8)	246	(17.7)
	35–39	1015	347	(34.2)	210	(20.7)	130	(12.8)	147	(14.5)	181	(17.8)
	≥40	245	95	(38.8)	47	(19.2)	29	(11.8)	32	(13.1)	42	(17.1)
BMI (kg/m^2^)	< 18.5	230	79	(34.3)	42	(18.3)	29	(12.6)	35	(15.2)	45	(19.6)
	18.5–24.9	2752	979	(35.6)	556	(20.2)	334	(12.1)	413	(15.0)	470	(17.1)
	25.0–29.9	731	273	(37.3)	172	(23.5)	93	(12.7)	78	(10.7)	115	(15.7)
	≥30.0	184	78	(42.4)	31	(16.8)	18	(9.8)	25	(13.6)	32	(17.4)
Smoking	No	3136	1101	(35.1)	636	(20.3)	392	(12.5)	459	(14.6)	548	(17.5)
	Yes	366	128	(35.0)	74	(20.2)	47	(12.8)	52	(14.2)	65	(17.8)
Multiple births	No	3631	1371	(37.8)	760	(20.9)	445	(12.3)	482	(13.3)	573	(15.8)
	Yes	417	101	(24.2)	71	(17.0)	43	(10.3)	88	(21.1)	114	(27.3)
PROM	No	2568	744	(29.0)	454	(17.7)	330	(12.9)	439	(17.1)	601	(23.4)
	Yes	1480	728	(49.2)	377	(25.5)	158	(10.7)	131	(8.9)	86	(5.8)
Placenta previa	No	3783	1425	(37.7)	803	(21.2)	443	(11.7)	501	(13.2)	611	(16.2)
	Yes	265	47	(17.7)	28	(10.6)	45	(17.0)	69	(26.0)	76	(28.7)
HDP	No	3925	1430	(36.4)	810	(20.6)	465	(11.8)	553	(14.1)	667	(17.0)
	Yes	123	42	(34.1)	21	(17.1)	23	(18.7)	17	(13.8)	20	(16.3)
GDM	No	3897	1435	(36.8)	806	(20.7)	470	(12.1)	542	(13.9)	644	(16.5)
	Yes	151	37	(24.5)	25	(16.6)	18	(11.9)	28	(18.5)	43	(28.5)
GA (weeks), mean (SD)			29.9	(3.2)	29.0	(3.3)	28.1	(3.4)	27.9	(3.1)	26.0	(2.7)

*BMI* body mass index, *GA* gestational age on admission, *GDM* gestational diabetes mellitus, *HDP* hypertensive disorders of pregnancy, *PROM* premature rupture of membrane

The overall proportion of patients receiving any maternal glucocorticoids during hospitalization was 46.2% (*n* = 1869). The frequency of repeated courses 14 days or later from the first course of glucocorticoids was 1.1% (*n* = 21) and 44.6% (*n* = 1805) of patients had received intravenous magnesium sulfate and ritodrine hydrochloride concurrently.

Table 2 shows the percentage of patients who had received maternal glucocorticoid in the recommended dose within 7 days or 14 days before preterm birth (< 34 weeks of gestation) during hospitalization according to duration of ritodrine hydrochloride infusion. Overall, only 23.2 and 27.9% of patients received glucocorticoids with recommended doses within 7 days and 14 days, respectively. In particular, the lowest percentage of patients who had received glucocorticoids as recommended was those who had received tocolytic treatment for ≥28 days.

**Table 2:** Crude outcome: administration of recommended dosage of antenatal glucocorticoid

Completed administration of glucocorticoid^a^	Total	Duration of ritodrine hydrochloride infusion				
		≤48 h	3–6 days	7–13 days	14–27 days	≥28 days
within 7 days	23.2% (939/4048)	25.0% (368/1472)	45.4% (377/831)	16.0% (78/488)	9.6% (55/570)	8.9% (61/687)
within 14 days	27.9% (1129/4048)	25.7% (378/1472)	46.9% (390/831)	40.4% (197/488)	15.6% (89/570)	10.9% (75/687)

^a^Intramuscular glucocorticoid injection of betamethasone 24 mg or dexamethasone 24 mg before preterm birth (< 34 weeks of gestation)

Table 3 shows the distribution of duration of tocolytic infusion among women who had received antenatal glucocorticoids within 7 days of delivery, stratified by gestational age at delivery. There were also lower percentages of patients who received glucocorticoids as recommended among patients who received long-term tocolysis (> 7 days).

**Table 3:** Duration of tocolytic infusion among women who received antenatal glucocorticoids within 7 days of delivery, stratified by gestational age at delivery

Gestational age at birth (weeks)	Total	Duration of ritodrine hydrochloride infusion				
		≤48 h	3–6 days	7–13 days	14–27 days	≥28 days
22–23	43	20.9% (9/43)	55.8% (24/43)	23.3% (10/43)	0	0
24–27	172	32.6% (56/172)	47.7% (82/172)	9.3% (16/172)	8.1% (14/172)	2.3% (4/172)
28–31	345	38.8% (134/345)	41.7% (144/345)	6.4% (22/345)	4.6% (16/345)	8.4% (29/345)
32–33	379	44.6% (169/379)	33.5% (127/379)	7.9% (30/379)	6.6% (25/379)	7.4% (28/379)
Total	939	368	377	78	55	61

The intervals between admission and maternal glucocorticoid administration were 1 (1–4) days and 1 (1–3) days (medians and interquartile ranges) in women who had received any maternal glucocorticoids and women who had received maternal glucocorticoids as recommended, respectively. The intervals between the last dose of maternal glucocorticoid and preterm birth were 3 (1–9) days and 2 (1–4) days (medians and interquartile ranges) in women who had received any maternal glucocorticoids and women who had received maternal glucocorticoids as recommended, respectively.

The results of multivariable logistic regression analysis of maternal glucocorticoid treatment as recommended within 7 days of delivery are presented in Table 4. After adjustment for variables, the rate of patients who received glucocorticoids as recommended was significantly associated with duration of ritodrine hydrochloride infusion. The odds ratio for receiving glucocorticoids as recommended was 2.52 (95% Confidence Interval [CI] 2.1, 3.1), 0.66 (95% CI 0.49, 0.89), 0.38 (95% CI 0.27, 0.53), and 0.37 (95% CI 0.26, 0.52) for women who received tocolytic treatment for 3–6 days, 7–13 days, 14–27 days, and ≥ 28 days, respectively. Obstetric complications such as multiple births, placenta previa, hypertensive disorders of pregnancy and gestational diabetes mellitus were not associated with the rate of patients who received glucocorticoids as recommended.

**Table 4:** Results of multivariable logistic regression for maternal glucocorticoid treatment as recommended within 7 days of delivery

	Odds ratio	95% Confidence interval	*P* value
Age (years)			
≥ 40	1.11	0.71, 1.75	0.65
35–39	1.13	0.80, 1.60	0.48
30–34	1.12	0.81, 1.57	0.50
25–29	0.95	0.67, 1.35	0.78
< 20	1.08	0.55, 2.10	0.83
20–24 (reference)	1.00		
BMI (kg/m^2^)			
≥ 30.00	1.34	0.91, 1.99	0.14
25.0–29.9	0.84	0.67, 1.05	0.12
< 18.5	0.97	0.67, 1.41	0.89
18.5–24.9 (reference)	1.00		
Smoking			
Yes	0.96	0.73, 1.28	0.79
No	1.00		
Multiple births			
Yes	1.06	0.78, 1.43	0.72
No	1.00		
PROM			
Yes	2.02	1.68, 2.41	< 0.001
No	1.00		
Placenta previa			
Yes	0.89	0.58, 1.35	0.57
No	1.00		
HDP			
Yes	0.97	0.57, 1.67	0.92
No	1.00		
GDM			
Yes	0.74	0.45, 1.22	0.23
No	1.00		
Gestational age at admission	0.99	0.97, 1.02	0.65
Duration of ritodrine hydrochloride			
≤ 48 h (reference)	1.00		
3–6 days	2.52	2.05, 3.10	< 0.001
7–13 days	0.66	0.49, 0.89	< 0.001
14–27 days	0.38	0.27, 0.53	< 0.001
≥ 28 days	0.37	0.26, 0.52	< 0.001

*BMI* body mass index, *GA* gestational age on admission, *GDM* gestational diabetes mellitus, *HDP* hypertensive disorders of pregnancy, *PROM* premature rupture of membranes

## Discussion

In the current study, 46.2% of patients received maternal glucocorticoids in various doses and with varying timing during hospitalization, whereas, only one-fourth of the 4048 women who had threatened preterm birth treated with ritodrine hydrochloride and delivered before 34 weeks of gestation received glucocorticoids as recommended. Only 1.1% of participants received repeat courses 14 days or later after their first course of glucocorticoids. Patients who received tocolytic treatment for ≥28 days were least likely to receive glucocorticoids as recommended.

Data have been lacking on the percentage of patients who give birth at less than 34 weeks of gestation in Japan and receive glucocorticoids as recommended, although this treatment is a proposed performance measure [9]. A previous retrospective cohort study in Canada reported that 2721/6870 patients (40%) received glucocorticoids as recommended 1–7 days before giving birth [16]. Another prospective cohort study in Ireland reported that 65/86 (76%) of women who delivered at < 34 weeks of gestation received glucocorticoids as recommended [17]. In comparison, the proportion of such women receiving antenatal corticosteroids as recommended is relatively low in Japan. The Japanese guidelines for obstetric physicians have recommended administration of antenatal corticosteroids for patients who were at risk of birth at less than 34 weeks of gestation since 2010 [12, 13]. There has been no change in the Japanese perinatal guidelines about this recommendation in the past 7 years.

In the present study, there was a correlation between a long delay between exposure to glucocorticoids and delivery and duration of tocolytic infusion, indicating that women who do not deliver within 7 days of glucocorticoids administration are exposed to tocolytic infusion for longer periods. In the current study, the multivariable logistic regression analysis of maternal glucocorticoid treatment was adjusted for the effects of measured confounders; however, some unmeasured confounders such as maternal vital signs and results of echography were not taken into account. The duration of hospitalization can also affect outcomes. Additionally, the duration of tocolytic infusion potentially varies in accordance with delay between glucocorticoids administration and delivery. We therefore concluded that the odds ratio in Table 4 does not indicate a causal relationship between long-term tocolysis and suboptimal maternal glucocorticoid treatment, but merely a variable associated with suboptimal maternal glucocorticoid administration during hospitalization. According to logistic regression analysis, premature rupture of membranes was also significantly associated with optimal maternal glucocorticoid administration. This association suggests that women with premature rupture of membranes on admission are more likely to receive early maternal glucocorticoid treatment because they are assessed appropriately by physicians.

In many developed countries, long-term tocolysis with intravenous tocolytic agents is uncommonly or never administered [2, 18]. Previous studies have suggested that long-term tocolysis is not justifiable because it is not effective in preventing preterm birth and tocolytic agents can be responsible for maternal complications. The present study has added a new finding, namely a negative association between long-term tocolysis and administration of maternal glucocorticoids as recommended.

Several limitations of this study should be acknowledged. First, it was a retrospective observational study based on an administrative database and therefore lacked some relevant clinical information, such as vital signs, parity, uterine cervical length according to ultrasound, vaginal microorganisms, and blood test findings. Second, there are no relatively small hospitals or clinics in the database we used. However, such hospitals rarely handle patients who deliver at < 34 weeks gestation because they tend to transfer women at high risk of preterm birth to larger centres. Third, because we excluded women who delivered at term from the current study, we were unable to analyze the potential overuse of antenatal glucocorticoids in all women with a diagnosis of threatened preterm birth who received tocolytics. Finally, the negative association between duration of tocolytics and suboptimal use of antenatal corticosteroids identified in the current study is potentially a result of bias introduced by unmeasured confounders. Additionally, our study design did not allow us to clarify whether the relationship between duration of tocolytics and suboptimal use of antenatal corticosteroids is causal. Future studies are warranted to evaluate a possible causal relationship between long-term tocolysis and use of antenatal corticosteroids, including in women who give birth at term.

## Conclusions

In conclusion, we demonstrated that many patients who receive tocolytic treatment for threatened preterm birth do not receive glucocorticoids as recommended in the present study. Additionally, we found that women with longer durations of ritodrine hydrochloride infusion are significantly less likely to receive optimal glucocorticoid treatment. Recommended treatment based on apparent evidences should be performed for the patients with threatened preterm birth in the real-world clinical setting.

## Abbreviations

CI: Confidence Interval
DPC: Diagnosis Procedure Combination
ICD-10: International Classification of Diseases Tenth Revision
SD: standard deviation
